# Kaempferol suppresses acetaminophen-induced liver damage by upregulation/activation of SIRT1

**DOI:** 10.1080/13880209.2021.1877734

**Published:** 2021-02-08

**Authors:** Mona Nasser BinMowyna, Nora Abdullah AlFaris

**Affiliations:** a College of Applied Medical Sciences, Shaqra University, Shaqra, Saudi Arabia; b Department of Physical Sport Science, Nutrition and Food Science, Princess Nourah Bint Abdulrahman University, Riyadh, Saudi Arabia

**Keywords:** APAP, oxidative stress, flavonoids, hepatic, apoptosis

## Abstract

**Context:**

Kaempferol, a flavonoid glycoside, has many hepatoprotective effects in several animals due to its antioxidant potential.

**Objective:**

This study evaluated the hepatoprotective effect of kaempferol against acetaminophen (APAP)-induced liver damage and examined whether the protection involved modulation of silent information regulator 1 (SIRT1) signalling.

**Materials and methods:**

Adult male Wistar rats were classified into four groups (*n* = 8) and treated as follows: control + normal saline (vehicle), control + kaempferol (250 mg/kg), APAP (800 mg/kg, a single dose) and APAP + kaempferol. Kaempferol was administered for the first seven days followed by administration of APAP. The study was ended 24 h after APAP administration.

**Results:**

At the histological level, kaempferol reduced liver damage in APAP-treated rats. It also reduced the hepatic levels of TNF-α (66.3%), IL-6 (38.6%) and protein levels of caspase-3 (88.2%), and attenuated the increase in circulatory serum levels of ALT (47.6%), AST (55.8%) and γ-GT (35.2%) in APAP-treated rats. In both the controls and APAP-treated rats, kaempferol significantly increased the hepatic levels of glutathione (GSH) and superoxide dismutase, suppressed MDA and reactive oxygen species (ROS) levels, increased protein levels of Bcl-2 and downregulated protein levels of Bax and cleaved Bax. Concomitantly, it reduced the expression of CYP2E1, and the activity and protein levels of SIRT1. Consequently, it decreased the acetylation of all SIRT1 targets including PARP1, p53, NF-κB, FOXO-1 and p53 that mediate antioxidant, anti-inflammatory and anti-apoptotic effects.

**Discussion and conclusions:**

This study encourages the use of kaempferol in further clinical trials to treat APAP-induced hepatotoxicity.

## Introduction

Acetaminophen (APAP) overdose is associated with liver damage (Yan et al. 2018). In the USA alone, APAP-induced hepatotoxicity is responsible for 500 deaths and >80,000 emergency room visits per year (Budnitz et al. 2011). Currently, APAP-induced hepatic injury is a commonly used model to study liver injury in small rodents and is the gold-standard for investigating the mechanisms of APAP-induced toxicity and potential therapeutic options (Jaeschke and Ramachandran 2018).

Overproduction of reactive oxygen species (ROS), mitochondrial damage, inflammation, necrosis and apoptosis are the best-described mechanisms through which APAP induces liver injury (Yan et al. 2018). In a healthy liver, cytochrome P450 (CYP450) is the major enzyme that metabolises APAP to N-acetyl-p-benzoquinone (NAPQI), known as hepatotoxic metabolite (James et al. 2003). The latter is converted to a less toxic form through binding with cellular glutathione (GSH) (Yoon et al. 2016). However, high hepatic levels of APAP reduce the intracellular levels of GSH that eventually leads to oxidative stress, overproduction of ROS (Jaeschke and Ramachandran 2018) and liver apoptosis (Nagy et al. 2010; Yan et al. 2018).

Silent information regulator 1 (SIRT1) has many biological functions in most cells including the liver cells, such as stimulation of cell survival, boosting of antioxidants, and inhibition of apoptosis (Chong et al. 2012). These effects are mainly mediated by its NAD^+^-dependent deacetylase activity that deacetylates several transcription factors involved in inflammation (nuclear factor-κB (NF-κB)), antioxidant potential and cell survival (peroxisome proliferators-activated receptor-γ (PPAR-γ)), forkhead transcriptional factors (FOXO and p53) and mitochondrial biogenesis (PPAR-γ coactivator-1α (PGC-1α)) (Chong et al. 2012). In the last decades, several studies have demonstrated an important protective role for SIRT1 in the liver of animals following APAP toxicity (Wang et al. 2015; Rada et al. 2018). Further, the level/activity of SIRT1 is significantly reduced in human and rodent livers following APAP exposure, whereas pharmacological activation of SIRT1 is highly protective (Wojnarová et al. 2015; Rada et al. 2018). Therefore, it is conceivable that any drug, which can induce protection against liver toxicity, can be used as a therapeutic strategy against APAP-induced liver toxicity.

Studies have revealed a positive correlation between human nutrition and APAP hepatotoxicity, wherein several plant flavonoids and ingredients protect the liver of mammals from the toxic effect of APAP (Lv et al. 2013; Jyotsna 2016; Huang et al. 2017). Kaempferol is a plant-derived flavonoid glycoside that is abundant in many plants (Yang et al. 2015). Dietary plants containing kaempferol are reported to be functional foods that provide a wide range of protection against different organ-induced oxidative damage, and protects from various lethal disorders by increasing antioxidants and suppressing inflammation and apoptosis in various tissues including the brain, liver, kidney and the heart (Chen and Chen 2013; Ren et al. 2019). Regarding its hepatoprotective effects, kaempferol protects rodent livers from oxidative stress-induced injury in animal models of alcohol-, bromobenzene-, rifampicin-, carbon tetrachloride- and APAP-induced toxicity (Sanz et al. 1994; Shih et al. 2013; Tsai et al. 2018; Ren et al. 2019). In most of these studies, kaempferol mediated protection by preventing the decrease in GSH stores, increasing the level of antioxidants, suppressing lipid peroxidation and inhibiting cytochrome P450 2E1 (CYP2E1).

In a well-designed study, kaempferol protected the left ventricles of rats from ischemia–reperfusion (I/R)-induced oxidative stress and apoptosis through upregulation/activation of SIRT1, and subsequent reduction in oxidative stress and inflammation (Guo et al. 2015). However, whether kaempferol can protect against APAP-induced hepatic toxicity, and whether this protection involves modulation of SIRT signalling remains unknown. Therefore, we tested the hypothesis that short-term treatment with kaempferol protects rat livers from APAP-induced damage through upregulation/activation of SIRT1, and subsequent deacetylation of the major SIRT1 target genes.

## Materials and methods

### Drugs and chemicals

Acetaminophen (cat. no. A5000), kaempferol (cat. no. 60010) and nuclear-extraction kit (cat. no. NXTRACT) were from Sigma-Aldrich (Cambridge, UK). A protease inhibitor (cat. no. 5873) was purchased from Cell Signaling Technology (Boston, MA). ELISA kits for determining the levels of tumour necrosis factor-α (TNF-α) and interleukin-6 (IL-6) (cat. no. CSB-E11987r and cat. no. CSB-E17064h, respectively) were purchased from Cusabio Technology LLC. (Houston, TX). A fluorescent assay kit to measure the intracellular levels of ROS was purchased from Cell Biolabs (cat. no. STA-347, OxiSelect, San Diego, CA). A spectrophotometer-based kit for measuring total levels of GSH was purchased from Trevigen (cat. no. 7511-100-K; Gaithersburg, MD). An assay kit for measuring malondialdehyde (MDA) and 10× RIPA buffer (cat. no. ab118970; cat. no. 156034, respectively) were purchased from Abcam (Cambridge, UK). SIRT1 activity kit (cat. no. CY-1151V) was from Medical and Biological Laboratories (Nagoya, Japan). A protein determination kit was purchased from ThermoFisher Scientific (cat. no. 23225; Piscataway, NJ). The sources of all the primary antibodies used are listed in Table 1.

**Table 1.** Antibodies used in this study.

Target	Cat. no.	Manufacturer	Dilution	kDa
SIRT1	sc-74504	Santa Cruz Biotechnology	1:1000	120
PARP1	sc-8007	Santa Cruz Biotechnology	1:1000	110
CYP2E1	ab28146	Abcam	1:1000	50
Bax	2772	Call Signaling Technology	1:1000	20
Bcl-2	Sc-7382	Santa Cruz Biotechnology	1:1000	26
Cleaved caspase-3	9661	Cell Signaling Technology	1:500	19
MnSOD	ab13533	Abcam	1:1000	26
NF-κB p65	ab16502	Abcam	1:1000	65
P53	Sc-126	Santa Cruz Biotechnology	1:1000	53
FOXO-1	2880	Cell Signaling Technology	1:1000	80
Lamin B1	ab65986	Abcam	1:1000	70
β-actin	4967	Cell Signaling Technology	1:2000	45
Acetyl lysine	9441	Cell Signaling Technology	1:500	

### Animals and ethical consideration

Healthy adult male Wistar albino rats (230 ± 5 g, 8 weeks old) were used in this study. All rats were provided from the animal house at the College of Pharmacy at King Saud University, Riyadh, Saudi Arabia. During the adaptation period (one week) and through the rest of the study period, all the rats were housed in plastic cages (temperature (21 ± 2 °C), humidity (60–70%) and 12 h light/dark cycle), fed normal chow and allowed free access to drinking water. All the experimental protocols were approved by the official Review Board at Princess Nourah University, Riyadh, Saudi Arabia (IRB number: 20-0091).

### Experimental design

The rats (*n* = 8/group) were classified into the following groups (1) control untreated group: rats were administered normal saline (NS) (vehicle) orally through the entire experimental period; (2) kaempferol-treated group: healthy rats were administered kaempferol orally (250 mg/kg; dissolved in NS) for seven days. (3) APAP-treated group: rats were administered NS for the first seven days and then a single dose of APAP (800 mg/kg, i.p.) at the end of day 7 to induce hepatic damage based on previous reports (Hong et al. 2012; El-Sayed et al. 2015); (4) kaempferol + APAP-treated group: rats were treated with kaempferol (250 g/kg, orally) for the first seven days and then administered a single dose of APAP (800 mg/kg, i.p) at the end of day 7. The dose of kaempferol used in this study was based on a previous report (Tsai et al. 2015) according to which kaempferol protects from acute APAP-induced liver damage at this concentration by suppressing CYP2E1. APAP was always prepared in warm NS (40 °C) (El-Sayed et al. 2015).

### Blood sampling and tissue collection

The rats were deeply anaesthetized with 1% sodium pentobarbital (single dose, 50 mg/kg, i.p) 24 h post-APAP administration and 2 mL of blood was collected from each rat by cardiac puncture into plain tubes to collect sera (1000 g/10 min) and stored at −20 °C until further usage. Then, all the rats were ethically euthanized and the livers were collected. Sections of the livers were fixed in 10% formalin for 24 h. Liver samples were also stored at −70 °C for biochemical and molecular assays.

### Preparation of tissue homogenates and cell fractions

To prepare total homogenates for biochemical assays, liver samples (50 mg) were homogenized in an appropriate volume of PBS (0.5 mL; pH = 7.4) and then centrifuged at 1000×*g* for 10 min. The supernatant was collected and stored at −20 °C until use. For western blotting, total liver homogenates were prepared by homogenizing 50 mg of the liver tissue in 0.5 mL 1× RIPA buffer containing 5 µL protease inhibitor followed by centrifugation at 10,000×*g* at 4 °C for 10 min. The supernatants were collected and frozen at −70 °C until use. The cytoplasmic/nuclear fraction was isolated using the NXTRACT kit following the manufacturer’s instructions, and the purity of the fractions was confirmed by western blotting for specific cytoplasmic and nuclear proteins in each fraction (β-tubulin and lamin B, respectively). Protein concentrations in the total and nuclear fractions of all samples were determined using a protein measurement kit.

### Determination of biochemical markers in serum and liver

Serum levels of major liver injury enzymes (markers) including gamma-glutamyl transferase (γ-GT), alanine aminotransferase (ALT) and aspartate aminotransferase (AST) were determined using an automatic-analyzer (Cobas^®^ 8000, Roche Diagnostics, Mannheim, Germany). Hepatic levels of GSH, MDA, ROS/RNS, MnSOD, TNF-α and IL-6 were measured using the supplied assay or ELISA kits as per the manufacturer’s instructions.

### Measurement of SIRT1 nuclear activity

Nuclear deacetylase activity of SIRT1 was measured using a SIRT1 activity assay kit with 25 µg of nuclear extract for each sample following the supplier’s instruction. The Cyclex SIRT1/Sir2 deacetylase assay kit relies on the deacetylase activity of SIRT1 on certain peptidases which when deacetylated cleave a fluorophore peptide substrate that results in a fluorescent signal which can be read every 2 min using a microplate fluorescence reader (FL600, Bio-Tek Instruments Inc., Winooski, VT) (excitation 340/emission 460 nm).

### Immunoprecipitation of NF-κB, FOXO-1, p53 and PGC-1α

Nuclear protein sample (100 µg) from each rat was diluted with 500 µL of HEPES buffer containing 10 µL protease inhibitor. Then, 20 µL 50% protein-A/G plus-agarose (diluted in lysis buffer) was added to each tube, followed by the addition of 2 µg rabbit IgG (diluted in lysis buffer). The tubes were placed on a shaker for 24 h at 4 °C and then centrifuged at 1000×*g* for 15 min at 4 °C to collect the supernatant. Antibodies (4 µg) against normal rabbit IgG or NF-κB p65, FOXO-1, p53 and PGC-1α were added to each supernatant. All tubes were then kept in a dark refrigerator at 4 °C for 2 h with shaking. Then, protein-A/G plus-agarose (50%, 30 µL) was added to each tube and the samples were incubated for 1 h at 4 °C, followed by washing with lysis buffer (500 µL) and centrifugation at 1200×*g* for 5 min at 4 °C. The precipitated complexes were collected from each sample, Laemmli buffer (30 µL; 2×) was added, and the sample was boiled for 5 min. All the samples were then cooled and used for western blotting for the detection of acetylated protein. The detection of the acetylated forms of each of the targets was performed using an acetyl lysine antibody (Table 1).

### Western blotting

Equal amounts of protein samples from the desired fractions and those collected from immunoprecipitation (40 µg) were separated by sodium dodecyl sulphate–polyacrylamide gel electrophoresis (SDS-PAGE) (8–15%) and transferred onto nitrocellulose membranes. All membranes were blocked, washed thrice (10 min/wash), with tris-buffered saline (TBS) containing Tween-20 (TBST), and immunoblotted with the primary antibody (prepared in TBST) at room temperature for 2 h with rotation (Table 1). Then, all the membranes were washed again with TBST buffer and incubated with the secondary antibody (prepared in TBST) for another 2 h at room temperature (with rotation). Protein bands were detected a Pierce ECL kit (ThermoFisher, Piscataway, NJ). The bands were photographed and their intensities were analysed using a C-Di Git blot scanner (LI-COR, Lincoln, NE).

### Histopathological studies

Formaldehyde fixed liver samples were re-dehydrated in ascending (70–100%) concentrations of ethanol (70–100%) and then stained using haematoxylin and eosin (H&E).

### Statistical analysis

All data were analysed using GraphPad Prism (version 8, Sydney, Australia) and are presented as mean ± SD. Normality test was initially performed for all data using the Kolmogorov–Smirnov test. The level of significance (*p* ˂ 0.05) among the data was analysed using the Kruskal–Wallis non-parametric analysis and Dunn’s *post hoc*.

## Results

### Kaempferol decreases liver injury and inflammation in APAP-treated rats

**Figure 1.**
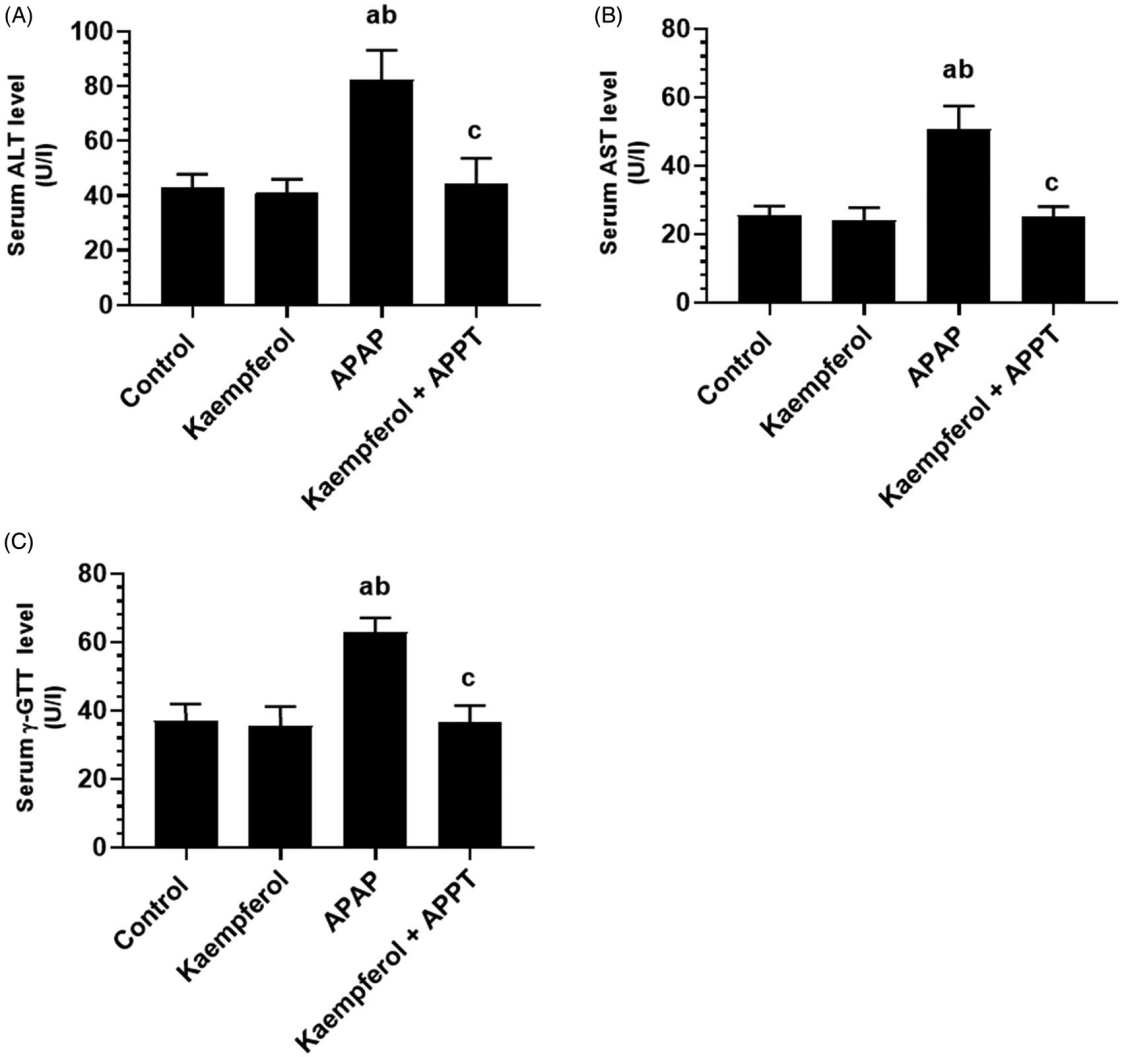
Circulatory levels of alanine aminotransferase (ALT), aspartate aminotransferase (AST) and γ-glutamyl transferase (γ-GT). Serum levels of ALT (A), AST (B) and γ-GT (C) in the experimental groups of rats were determined using an automatic-analyser. Values are expressed as mean ± standard deviation (SD; eight rats/group). ^a^vs. control group. ^b^vs. kaempferol-treated control group. ^c^vs. APAP (acetaminophen)-treated rats.

**Figure 2.**
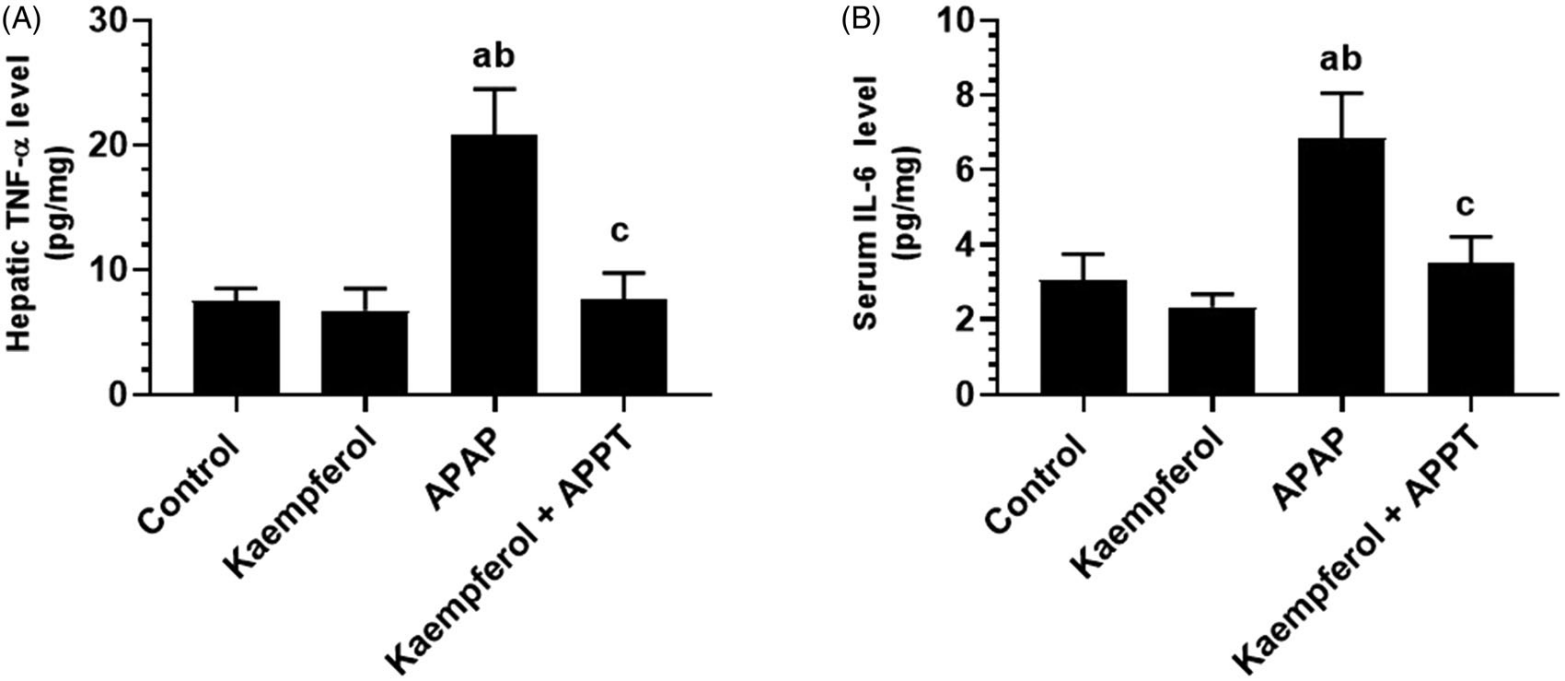
Levels of tumour necrosis factor-α (TNF-α) (A) and interleukin 6 (IL-6) (B) in the liver homogenates of the experimental groups. Values are expressed as mean ± SD (eight rats/group). ^a^vs. control group. ^b^vs. kaempferol-treated control group. ^c^vs. APAP (acetaminophen)-treated rats.

Circulatory levels of ALT, AST and γ-GT, and hepatic levels of TNF-α and IL-6 were significantly higher in APAP-treated group compared to all the other groups (Figures 1(A–C) and 2(A,B)). However, the administration of kaempferol to healthy rats (kaempferol-treated control rats) did not affect any of these markers (Figures 1(A–C) and 2(A,B)). In contrast, levels of all these biochemical markers were significantly suppressed in kaempferol + APAP-treated rats in contrast to APAP-treated rats (Figures 1(A–C) and 2(A,B)).

### Kaempferol suppresses hepatic oxidative stress in APAP-treated rats

**Figure 3.**
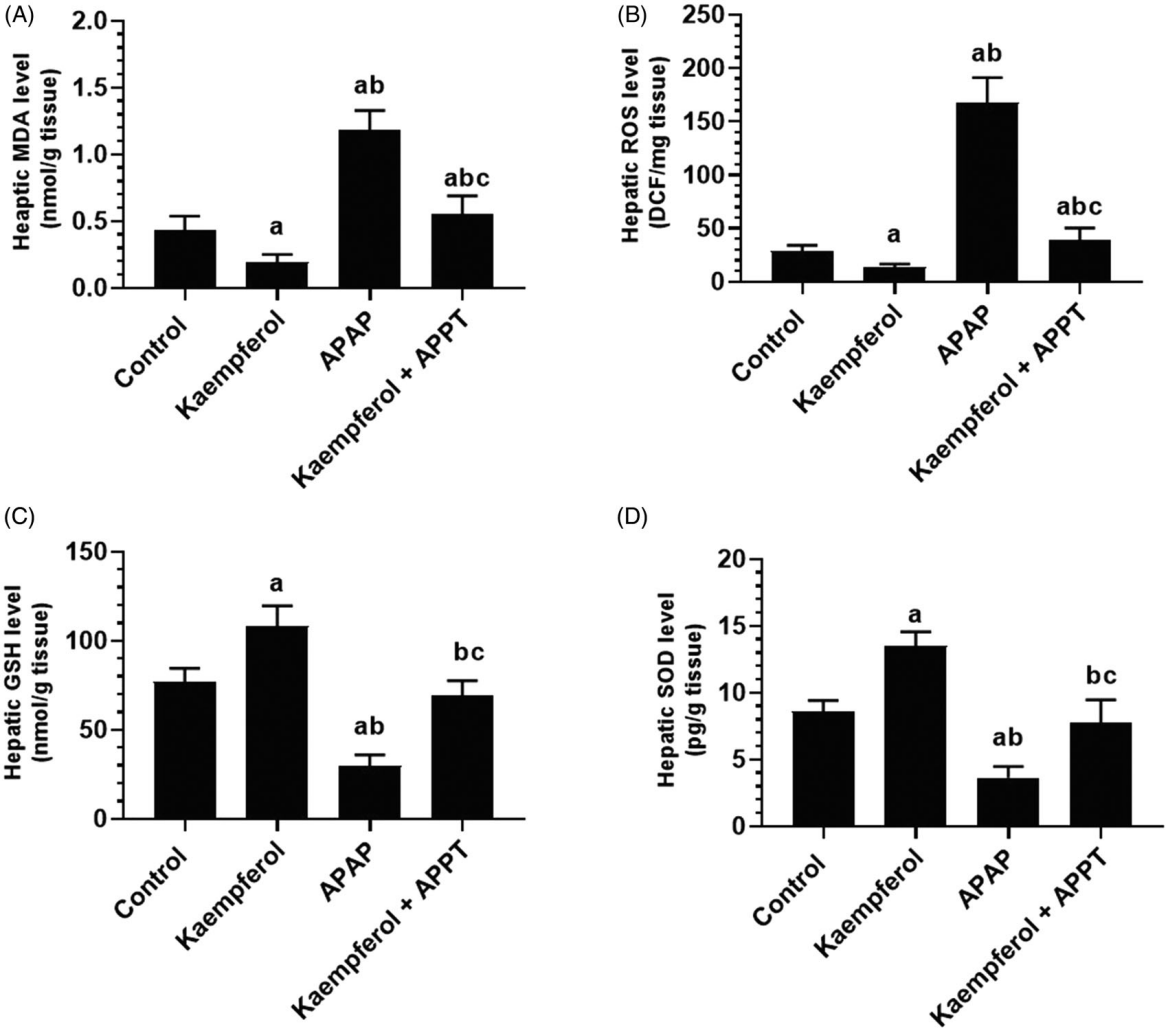
Levels of malondialdehyde (MDA) (A), reactive oxygen species (ROS) (B), glutathione (GSH) (C) and superoxide dismutase (SOD) (D) in the liver homogenates of the experimental groups. Values are expressed as mean ± SD (eight rats/group). ^a^vs. control group. ^b^vs. kaempferol-treated control group. ^c^vs. APAP (acetaminophen)-treated rats.

APAP-treated rats showed significantly higher hepatic levels of ROS and MDA, and lower levels of GSH and SOD compared to rats of all the other groups (Figure 3(A–D)). Administration of kaempferol to healthy control or APAP-treated rats significantly boosted the endogenous levels of GSH (45.3 and 143%, respectively) and SOD (64.7 and 128.3%, respectively), and inhibited the intracellular levels of ROS and MDA (Figure 3(A–D)).

### Kaempferol improved liver architecture in APAP-treated rats

**Figure 4.**
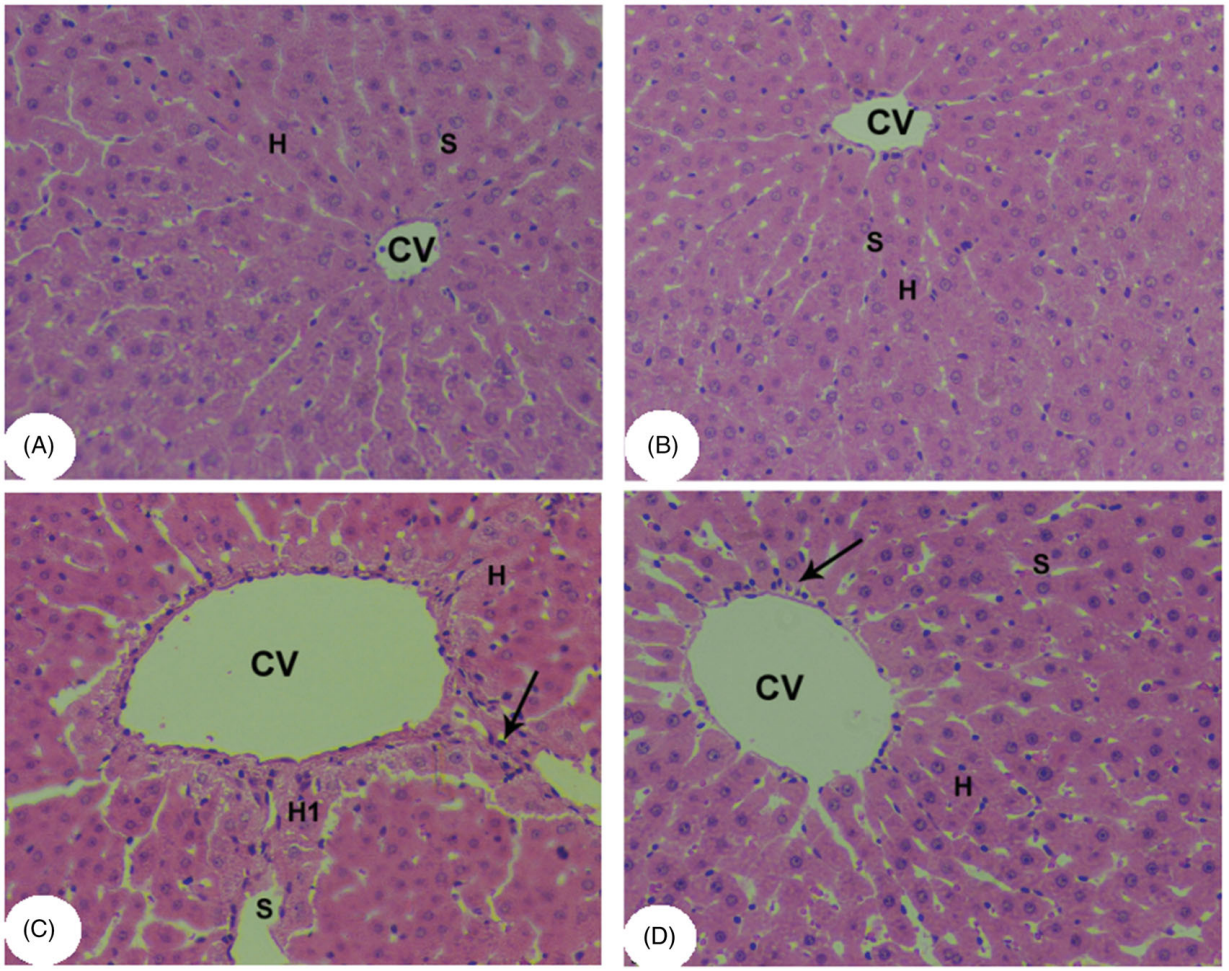
Light micrographs of liver tissues from all groups after H&E staining. (A, B) control and kaempferol-treated rats, respectively, showing normal hepatocytes (H) and blood sinusoids (S) around the central veins (CVs). (C) APAP-treated rats showing swollen hepatocytes and wide blood sinusoids around the CVs. Some apoptotic and necrotic hepatocytes (H1), and inflammatory cells (arrows) were also seen. Some accumulation of erythrocytes was observed inside the CVs. (D) Kaempferol + APAP-treated rats showing normal hepatocytes and blood sinusoids around the CVs. Very few inflammatory cells (arrows) were seen around the CVs.

Normal liver architecture with intact hepatocytes, central vein (CV) and sinusoids were seen in both control and kaempferol-treated control rats (Figure 4(A,B)). However, the APAP-treated rats showed degeneration and swelling of hepatocytes with the presence of inflammatory cell infiltration, and apoptotic and necrotic cell death (Figure 4(C)). However, close to normal features were observed in kaempferol + APAP-treated rats (Figure 4(D)).

### Kaempferol suppresses haptic apoptosis in APAP-treated rats by upregulation of Bcl-2 and MnSOD

**Figure 5.**
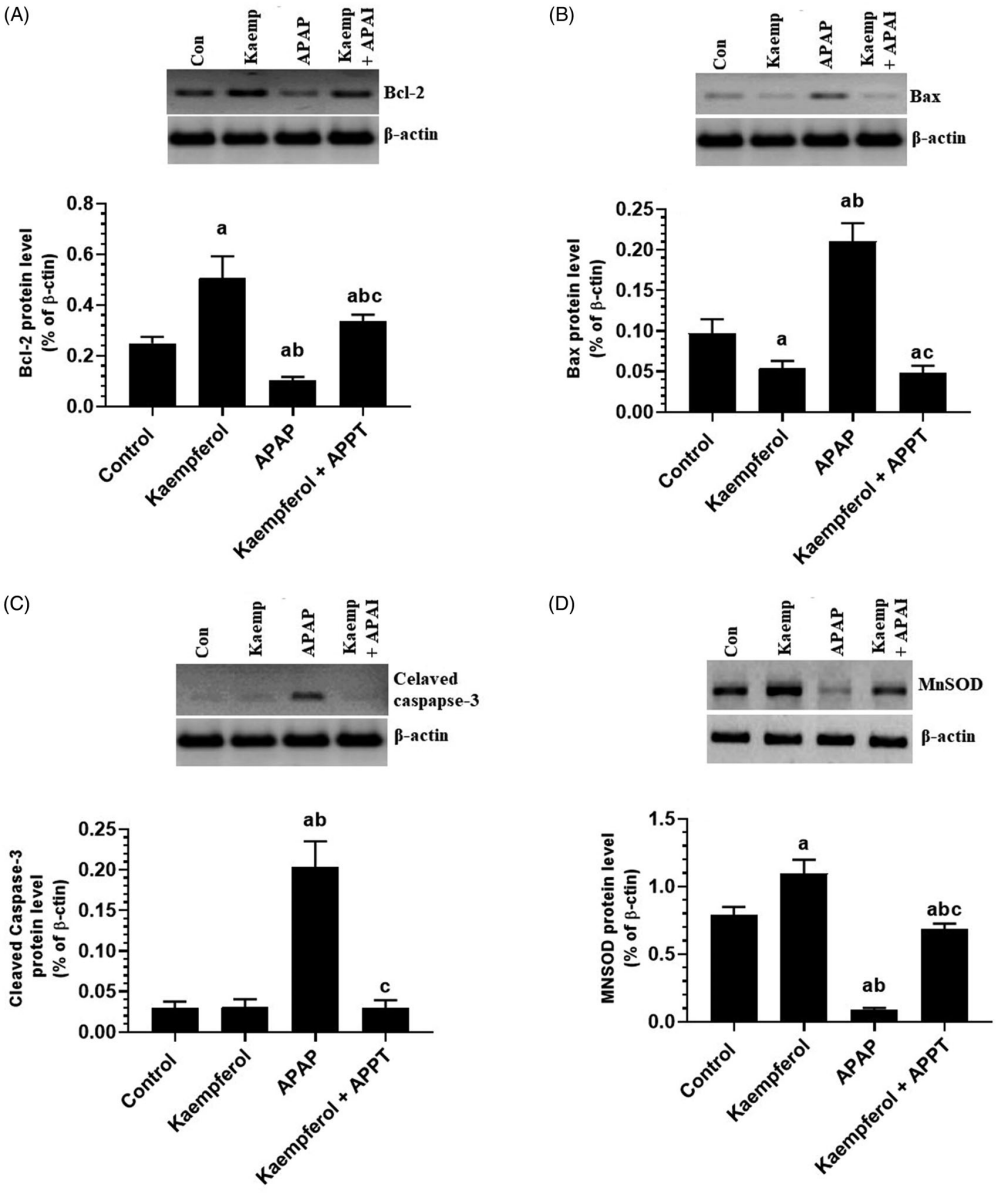
Protein levels of Bcl-2 (A), Bax (B), cleaved caspase-3 (C) and manganese superoxide dismutase (MnSOD) (D) in the liver homogenates of all experimental groups. Values are expressed as mean ± SD (six rats/group). ^a^vs. control group. ^b^vs. kaempferol-treated control group. ^c^vs. APAP (acetaminophen)-treated rats.

APAP significantly increased the protein levels of Bax and cleaved caspase-3, but downregulated those of Bcl-2 and MnSOD in the liver of treated rats compared to that in healthy untreated rats (Figure 5(A–D)). However, pre-treatment of the APAP-treated rats with kaempferol reversed these alterations and restored them to their control levels (Figure 5(A–D)). Of note, treatment of healthy control rats with kaempferol did not affect cleaved caspase-3 levels, but significantly upregulated hepatic levels of Bcl-2 and MnSOD compared to the control untreated rats (Figure 5(A–D)).

### Kaempferol suppresses hepatic activity of CYP2E1 and PARP1, but upregulates hepatic SIRT1 in APAP-treated rats

**Figure 6.**
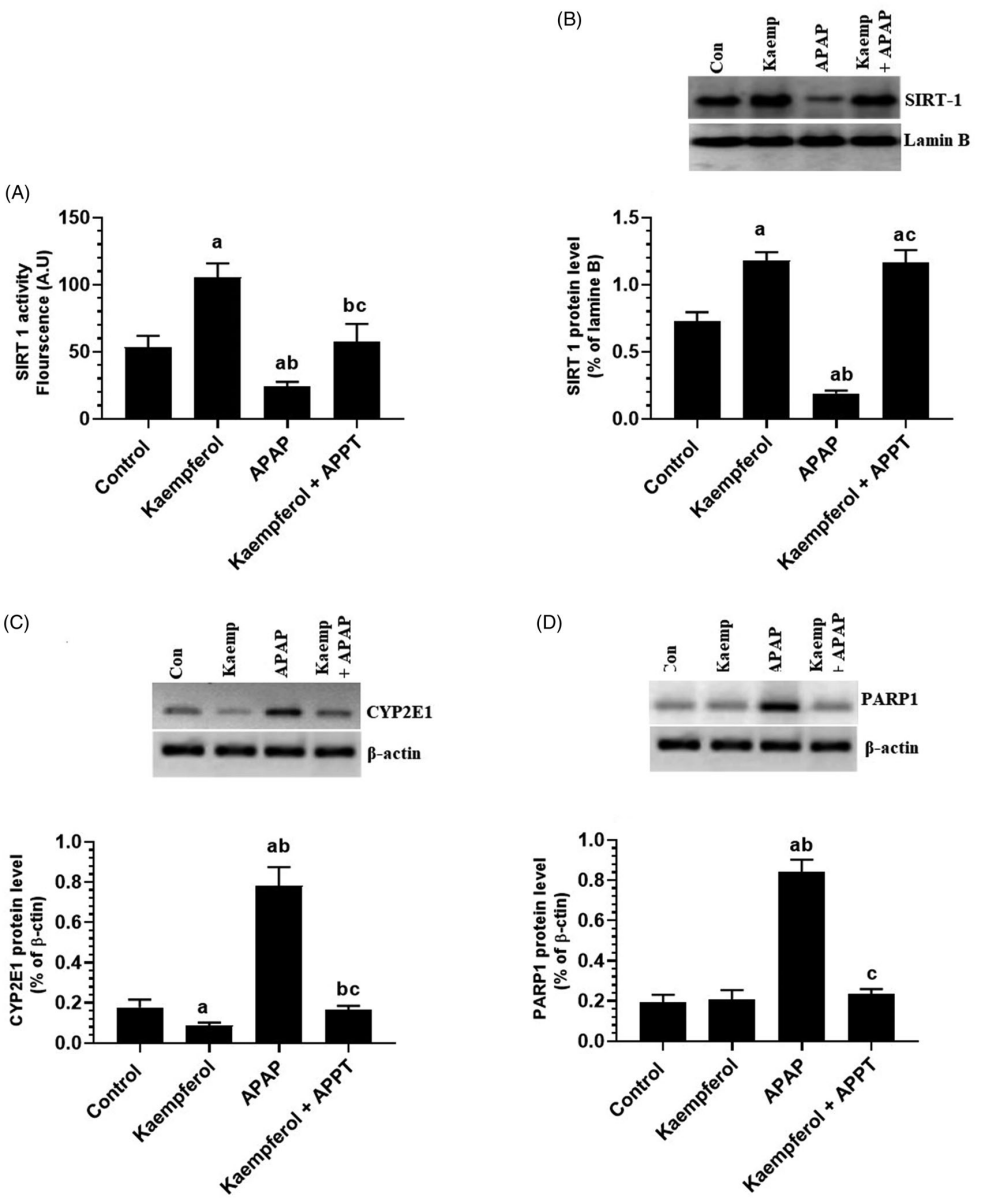
Nuclear activity of SIRT1 (A), protein levels of SIRT1 (B), cytochrome-2E1 (CYP2E1) (C) and PARP1 (D) in the liver homogenates of all experimental groups. Values are expressed as mean ± SD (six rats/group). ^a^vs. control group. ^b^vs. kaempferol-treated control group. ^c^vs. APAP (acetaminophen)-treated rats.

**Figure 7.**
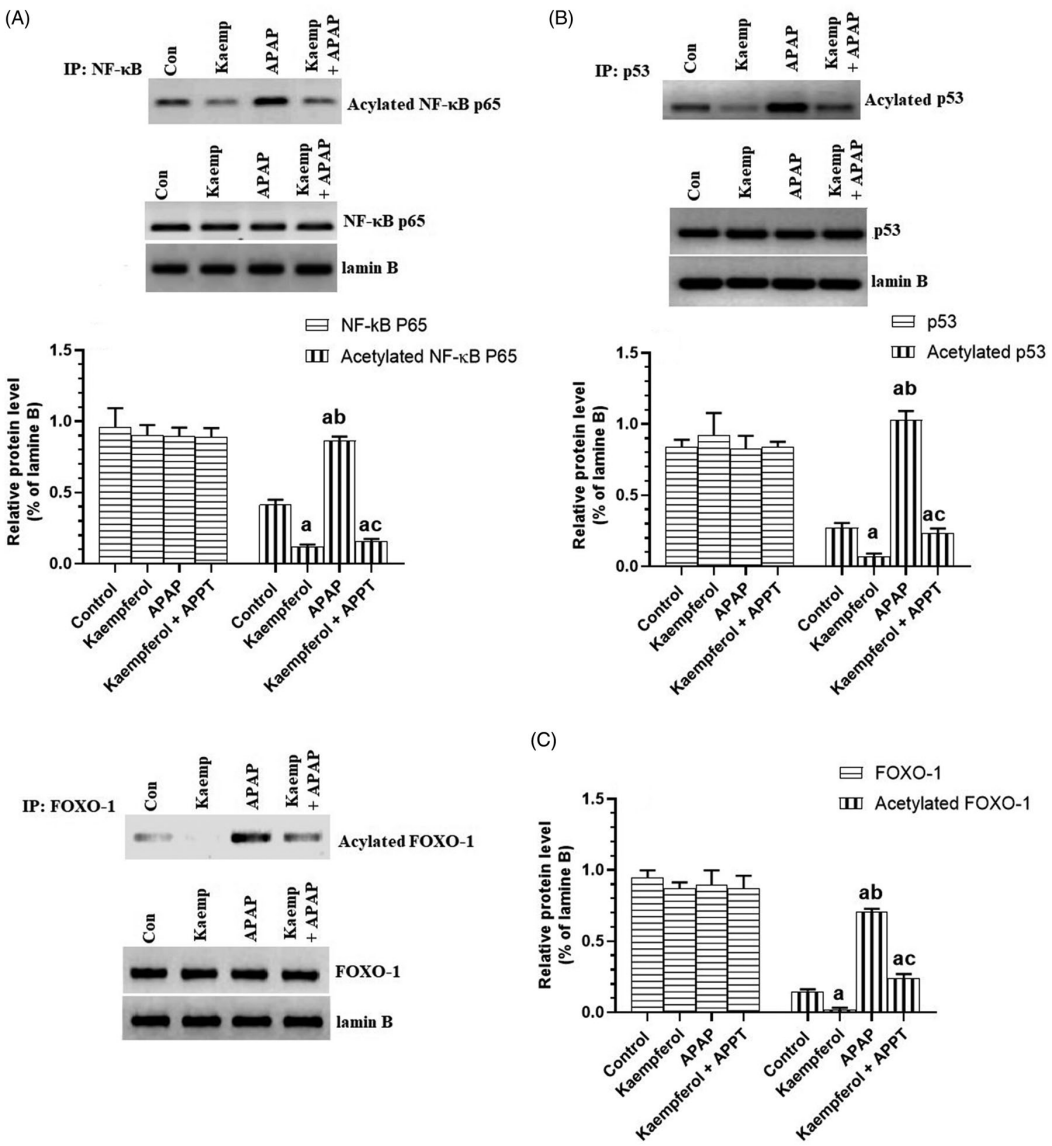
Total nuclear levels of NF-κB P65/acetylated NF-κB p65 (A), P53/acetylated p53 (B), FOXO-1/acetylated FOXO-1 (C) in the livers of all experimental groups. Values are expressed as mean ± SD (six rats/group). ^a^vs. control group. ^b^vs. kaempferol-treated control group. ^c^vs. APAP (acetaminophen)-treated rats.

Protein expression of CYP2E1 and PARP1, and nuclear-acetylation of NF-κB p65, p53 and FOXO-1 were significantly upregulated, while the nuclear level and activity of SIRT1 were significantly decreased in the livers of APAP-treated rats compared to the untreated rats (Figures 6(A–D) and 7(A–C)). These events were reversed by pre-treatment of the APAP-treated rats with kaempferol (Figures 6(A–D) and 7(A–C)). Interestingly, among all the measured parameters, the administration of kaempferol to control rats significantly increased the activity and nuclear levels of SIRT1 protein, and suppressed the protein level of CYP2E1 in the livers of untreated healthy rats (Figures 6(A–D) and 7(A–C)).

## Discussion and conclusions

Kaempferol, a dietary flavonoid, was previously shown to protect the liver of rodents from APAP overdose-induced oxidative stress and apoptosis through downregulation of CYP2E1 (Tsai et al. 2018). The data obtained from the present study confirmed this finding, and provided evidence for another independent mechanism of action. In this study, we provide the first evidence that the hepatoprotective effect of kaempferol in this animal model involves upregulation and activation of SIRT1, and subsequent deacetylation of the major signalling regulators, including p53, NF-κB and FOXO-1.

Previous studies have shown that a single dose of APAP at a concentration higher than 800 mg/kg is required to induce hepatotoxicity and liver damage in rats (Hong et al. 2012; El-Sayed et al. 2015). Such a dose is associated with early activation of both necrosis and apoptosis (Kon et al. 2004; Bajt et al. 2006; Nakagawa et al. 2008; Kaplowitz et al. 2015), thus supporting the argument that APAP overdose activates both necrosis and apoptosis in the liver of mammals. However, necrosis is usually associated with damage to the cell membrane, release of cellular contents (enzymes), and inflammation (Clemens et al. 2000; Vanden Berghe et al. 2006). Similarly, APAP-activated both necrosis and apoptosis in the liver of rats as shown by histological evidence, increase in liver enzymes and hepatic level of inflammatory cytokines, downregulation of Bcl-2, and upregulation of Bax and cleaved caspase-3. Consistent with these data, severe hepatic damage accompanied by increase in serum levels of ALT, AST and γ-GT, as well as higher levels of several inflammatory cytokines were previously observed in rodents after administration of APAP (Randle et al. 2008; Rasool et al. 2010; Aseervatham et al. 2014). Besides, APAP is well known to activate intrinsic apoptosis in liver cells, as well as in cultured hepatocytes by downregulation of Bcl-2 and concomitant mitochondria damage mediated by activation of Bax (Kon et al. 2004; Li et al. 2013; Wang et al. 2015; Tsai et al. 2018).

Pre-treatment of rats with kaempferol prior to administration of APAP attenuated the augmentation in serum level of liver damaging enzymes as well as that of hepatic inflammatory and apoptotic markers, and the livers of these rats showed almost normal architecture. This is our first evidence for the protective effect of kaempferol in such an animal model and is direct evidence that the hepatoprotective mechanism of kaempferol involves anti-inflammatory and anti-apoptotic effects. In support of this, a previous study showed that kaempferol prevents and attenuates APAP-induced liver damage through increasing the level of Bcl-2, suppressing Bax, and inhibiting inflammation (Tsai et al. 2018). In addition to the liver, kaempferol inhibited inflammation and prevented apoptosis in various tissues including the lungs, brain, peripheral muscles of different animal models of chemical-induced liver injury (Ruiz et al. 2006; Yu et al. 2013; Yang et al. 2015; Chitturi et al. 2019). The significant finding of this study is that kaempferol also stimulates Bcl-2 and inhibits Bax in the liver in control healthy rats, thus indicating a regulatory effect of kaempferol on these apoptotic mediators. As will be discussed later, these effects may be due to the ability of kaempferol to stimulate SIRT1 and subsequently deacetylate various transcription factors that play significant roles in preventing cell apoptosis.

Nonetheless, overproduction of ROS mediated by depletion of antioxidants (such as GSH) due to over-activation of CYP2E1 and subsequent increases in intracellular levels of NAPQI is the best-described mechanism of APAP-induced liver toxicity (Yoon et al. 2016; Yan et al. 2018). However, the antioxidant and hepatoprotective effects of kaempferol are well-confirmed in a variety of animal models in alcohol, bromobenzene, rifampicin, carbon tetrachloride and APAP-induced liver damage (Sanz et al. 1994; Shih et al. 2013; Wang et al. 2015; Tsai et al. 2018; Ren et al. 2019). In majority of these studies, the hepatoprotective effect of kaempferol is mediated by suppressing ROS, inhibiting lipid peroxidation, boosting endogenous antioxidants, and inhibiting CYP2E1. Along the same line of evidence, our data also suggest that the hepatoprotective effect of kaempferol in the APAP-induced rat model in this study involves all these mechanisms as shown by the data. Interestingly, kaempferol also suppressed ROS, lipid peroxidation, expression of CYP2E1, and upregulated MnSOD in the liver of control healthy rats. Based on these observations, we concluded that the antioxidant effect of kaempferol, in both healthy and APAP-treated rats, involves the downregulation of CYP2E1 and upregulation of MnSOD.

The antioxidant effects of kaempferol have been researched extensively and shown to be independent of the effects on CYP2E1. Kaempferol has a unique structure that contains several domains (oxo group at C4, a double bond at C2–C3, and numerous hydroxyl groups at C3, C5 and C4), all of which can scavenge free radicals at an IC_50_ of 0.5 µM (Randle et al. 2008; Calderón-Montaño et al. 2011). Besides, kaempferol scavenges superoxide radicals (O_2_.) and prevents the formation of hydroxyl radicals (OH^–^) by chelating several ions like ferrous and cuprous (Van Acker et al. 1996; Mira et al. 2002). Furthermore, kaempferol increases the expression of several antioxidant enzymes and GSH by upregulating their master regulator including nuclear Nrf-2, and the heme oxygenase-1 gene (Maridonneau-Parini 1986; Ozgova et al. 2003; Doronicheva et al. 2007; Hong et al. 2009; Calderón-Montaño et al. 2011; Zhang et al. 2019). Therefore, it is possible that kaempferol improved MnSOD and GSH in the livers of the control and APAP-treated rats by upregulation/activation of the Nrf-2/HO-1 axis. However, crosstalk exists between oxidative stress and inflammation, and these processes synergistically cause elevation in the levels of ROS and tissue damage (Dandekar et al. 2015). Since the levels of inflammatory markers remained unchanged, but levels of ROS were significantly decreased in the liver of control rats that were treated with kaempferol, it seems logical to conclude that the anti-inflammatory effect of kaempferol is secondary to its antioxidant potential.

On the other hand, it is well documented in rodent models that APAP suppresses the hepatic level of SIRT1 (Wang et al. 2015; Wojnarová et al. 2015). In this animal model, pharmacological activation of SIRT1 by resveratrol or CAY10591 afforded protection (Wang et al. 2015; Wojnarová et al. 2015). Similarly, genetic deletion of SIRT1 prevented APAP-induced liver apoptosis in mice (Rada et al. 2018). However, several cellular mechanisms regulating SIRT1 are still under investigation. Consistent with this, negative cross-talk between SIRT1 and NF-κB p65 was recently reported, where one inhibits the other (Kauppinen et al. 2013). In a well-designed study, activation of NF-kB pathway was shown to be the main mechanism by which APAP suppresses the hepatic expression of SIRT1, whereas blocking this pathway maintains SIRT1 level and prevents APAP-induced liver damage (Rada et al. 2018). However, oxidative DNA damage depletes level/activity of SIRT1 by depleting its substrate, NAD^+^, mediated by the activation of another NAD^+^-dependent DNA repair enzyme poly ADP-ribose polymerase-1 (PARP1) (Rajamohan et al. 2009). SIRT1 also deacetylates and negatively affects the activity and level of PARP1 (Rajamohan et al. 2009).

In most cells, SIRT1 is a pro-survival factor that promotes cell survival and proliferation, suppresses cell inflammation and oxidative stress, and promotes mitochondrial biogenesis and ATP synthesis by the deacetylation of numerous transcription factors (Chang and Guarente 2014). At the molecular level, SIRT1 inhibits P53 acetylation, thus inhibiting its nuclear translocation and subsequent synthesis of Bax (Vaziri et al. 2001). Also, SIRT1 deacetylates FOXO1 (in the nucleus), and thus suppresses the expression of many apoptotic genes (i.e., FasL and Bim) and increases the expression of antioxidant and anti-apoptotic genes (i.e., Bcl-2 and MnSOD) (Chong et al. 2012). Also, SIRT1 deacetylates and inactivates NF-κB p65, which normally stimulates inflammation by upregulation of inflammatory cytokines and induces apoptosis by upregulation of Bax and inhibition of Bcl-2 (Matsuzawa et al. 2002; Chong et al. 2012). Of note, a very recent study showed that melatonin-induced activation of SIRT1 protected against ethanol-induced liver damage mainly through downregulating of hepatic CYP2E1 (Lee et al. 2020), thus suggesting that the expression of CYP2E1 is controlled by SIRT1.

As expected, the liver of APAP-treated rats of this study showed low expression and activation of SIRT1 with a concomitant increase in the expression of CYP2E1, PARP1 and acetylation of p53, NF-κB p65 and FOXO-1. Hence, it is possible that over-activation of CYP2E1, in the liver of APAP-treated rats, led to the decrease in SIRT1 levels. Besides, it is possible that this decrease in SIRT1 level is mediated by activation of NF-κB and PARP1 due to the overproduction of ROS and DNA damage. Therefore, our data highlights a novel mechanism in which APAP suppresses hepatic level of SIRT1 through activating PARP1. Furthermore, our data suggest that APAP-induced acetylation of p53, FOXO-1 and NF-κB significantly contributes to increased level of Bax and decreased level of MnSOD, GSH and Bcl-2 in the liver of APAP-treated rats.

Nonetheless, the most interesting finding reported here is that kaempferol upregulated and activated SIRT1, downregulated CYP2E1 and PARP-1, and inhibited the acetylation of the above-mentioned transcription factors in the livers of APAP-treated rats. Similar results, with no effect on PARP1, have also been reported in the hepatic homogenates of control + kaempferol-treated rats. Hence, it can be concluded that the hepatoprotective effects of kaempferol are mediated by activation of SIRT1 and inhibition of PARP1. Hence, in addition to a possible activation of the Nrf2/HO-1 axis, it is also possible that kaempferol increased the levels of SOD and GSH, and stimulated the protein levels of Bcl-2 in the livers of the control and APAP-treated rats through SIRT1-induced deacetylation of FOXO-1. However, whether SIRT1 mediates the inhibition of CYP2E1 after kaempferol treatment cannot be inferred from these data and further experiments at the molecular level using SIRT1 inhibitors or siRNA are required to identify the exact mechanism of protection exerted by kaempferol.

Our study contributes some significant findings and shows that the hepatoprotective effect of kaempferol in an APAP-induced liver damage rat model involves two related mechanisms including inhibition of CYP2E1 and activation of SIRT1. However, the precise mechanism through which kaempferol activates SIRT1 needs further investigation.
